# Subclinical markers of cardiovascular disease predict adverse outcomes in chronic kidney disease patients with normal left ventricular ejection fraction

**DOI:** 10.1007/s10554-016-1059-x

**Published:** 2017-01-24

**Authors:** Samir Sulemane, Vasileios F. Panoulas, Athanasios Bratsas, Julia Grapsa, Edwina A. Brown, Petros Nihoyannopoulos

**Affiliations:** 1 Imperial College London, National Heart and Lung Institute, Sydney Street, London, SW6 3NP UK; 2 Imperial College Healthcare NHS, Hammersmith Hospital, Ducane road, London, W12 0HP UK; 3 Imperial College Renal and Transplant Centre, Hammersmith Hospital, Ducane road, London, W12 0HP UK

**Keywords:** Chronic kidney disease, Speckle tracking echocardiography, Aortic pulse wave velocity, Cardiovascular outcomes

## Abstract

Emerging cardiovascular biomarkers, such as speckle tracking echocardiography (STE) and aortic pulse wave velocity (aPWV), have recently demonstrated the presence of subclinical left ventricular dysfunction and arterial stiffening in patients with chronic kidney disease (CKD) and no previous cardiovascular history. However, limited information exists on the prognostic impact of these biomarkers. We aimed to investigate whether STE and aPWV predict major adverse cardiac events (MACE) in this patient population. In this cohort study we prospectively analysed 106 CKD patients with no overt cardiovascular disease (CVD) and normal left ventricular ejection fraction. Cardiac deformation was measured using STE while aPWV was measured using arterial tonometry. The primary end-point was the composite of all-cause mortality, acute coronary syndrome, stable angina requiring revascularization (either using percutaneous coronary intervention or coronary artery bypass surgery), hospitalization for heart failure and stroke. Over a median follow up period of 49 months (interquartile range 11–63 months), 26 patients (24.5%) reached the primary endpoint. In a multivariable Cox hazards model, global longitudinal strain (GLS) (HR 1.12, 95% CI 1.02–1.29, p = 0.041) and aPWV (HR 1.31, 95% CI 1.05–1.41, p = 0.021) were significant, independent predictors of MACE. GLS and aPWV independently predict MACE in CKD patients with normal EF and no clinically overt CVD.

## Introduction

Chronic kidney disease (CKD) patients are a group at significantly increased risk of cardiovascular (CV) events, as a result of prolonged exposure to an ever-growing array of traditional and non-traditional risk factors [1]. Most risk factors exert their influence on the myocardium and arterial wall itself, causing collateral damage to organs when circulatory function is altered sufficiently to result in clinical disease [2]. The process of circulatory damage occurs over many years, and although outcomes [such as stroke or myocardial infarction (MI)] occur at identifiable discrete times, it is much more difficult to cross-sectionally assess the influence of established biomarkers of CV risk, such as glucose or cholesterol, without some surrogate for cardiovascular outcomes [3]. In the last few years a number of surrogate markers of subclinical myocardial and arterial dysfunction have emerged to quantify the degree of subclinical CV impairment. These surrogate markers are particularly relevant to CKD, a population at increased risk of CV disease (CVD) [1].

Conventional echocardiographic measures of left ventricular (LV) geometry, structure and function are significant predictors of outcomes in the CKD population. Prevalence of LV hypertrophy (LVH) [4, 5], diastolic dysfunction (DD) and LV systolic impairment [6–9], predict adverse cardiovascular outcomes and increased and mortality. Although of importance the bulk of echocardiographic studies have been performed in the presence of major structural and functional cardiac abnormalities. Recently, speckle-tracking analysis, a novel echocardiographic technique that assesses intrinsic deformation (strain) of the myocardium has been proposed as a sensitive tool to detect myocardial dysfunction before a decrease in LV ejection fraction (LVEF) occurs [10]. Additionally, speckle-tracking echocardiography (STE) has been validated against sonomicrometry and cardiac magnetic resonance imaging (cMRI) [11]. Currently, some authors have used STE to investigate the predictive value of subclinical myocardial disease in both end stage renal failure (ESRF) [12] and CKD [13]. Although these studies provide some insight into the relationship between subclinical myocardial disease and CV mortality they are limited by the fact that novel elements of deformation, such as strain rate (SR) or LV twist, were not studied. In addition large proportions of the population had previous cardiac events, confounding the significance of STE parameters in asymptomatic patients without previous cardiovascular history.

Previous studies have also shown that aortic stiffness as measured by aortic pulse wave velocity (aPWV) predicts cardiovascular mortality in patients with CKD [14] and ESRF [15]. Furthermore, recent work demonstrated that arterial remodelling is associated with CKD progression [16] and that in advanced CKD, stiffness parameters can predict CKD progression [17]. Despite this, no prospective data is available analysing the role of aPWV in predicting major adverse CV events in CKD patients with normal LVEF.

The current study hypothesized that subclinical markers of myocardial (STE) and vascular (aPWV) disease can provide important prognostic value in predicting major adverse cardiac events (MACE) in CKD patients (of all stages) with no overt CVD and normal LVEF by conventional echocardiography.

## Methods

### Study population

A prospective, observational cohort study was conducted involving 121 consecutive patients, recruited from outpatient nephrology clinics at Hammersmith and Charing Cross Hospitals, Imperial College Healthcare NHS, London, UK. Written informed consent was obtained from all participants and the study was approved by the UK National Research Ethics Committee Service (REC 10/H0704/81). The study conforms to the principles outlined in the Declaration of Helsinki. Patients on haemodialysis, or those with clinical or echocardiographic evidence of LV systolic dysfunction (Simpson biplane LVEF < 55%), presence of regional wall motion abnormalities, significant valvular abnormalities (moderate or severe), presence of atrial fibrillation or flutter, known pulmonary hypertension, congenital heart disease, cardiomyopathy, pericardial disease, or inadequate echocardiographic acoustic windows were excluded from this study.

All patients were asymptomatic from a cardiovascular point of view, and had normal systolic function, as assessed by conventional echocardiography. We carefully reviewed the clinical notes for all patients and any patient displaying evidence or signs of heart failure was excluded from the study.

### Study protocol

CKD etiology, cardiovascular risk factors, and detailed drug history were recorded. Bio-chemical results were obtained from the most recent renal clinic review, provided that there was no evidence of superimposed acute kidney injury during the time of blood sampling. The value of estimated glomerular filtration rate (eGFR) was calculated using the four-variable equation in the Modification of Diet in Renal Disease (MDRD) study [18].

### Follow-up

All participants were followed up for a median time of 49 months (interquartile range 11–63 months). The primary end-point was a composite of all-cause mortality, acute coronary syndrome, stable angina requiring revascularization [either using percutaneous coronary intervention (PCI) or coronary artery bypass surgery (CABG)], hospitalization for heart failure and stroke. Deaths were identified from the office of national statistics (ONS). All patients were followed up either in outpatient clinics or by telephone conversation.

### Conventional echocardiography

All echocardiographic parameters were obtained using the VIVD 7 echocardiographic machine (GE healthcare, Little Chalfont, United Kingdom). The images obtained were stored and subsequently analyzed offline in EchoPac version 12 (GE Healthcare) by a single, accredited investigator blinded to all baseline data.

LVEF was obtained using the modified Simpson’s rule [19]. LV dimensions and LV wall thickness were measured in the parasternal long axis view [19]. Left atrial (LA) volume indexed to body surface area (LAVI) was measured using the LA disk summation algorithm [19]. LV mass was estimated using the Deveraux formula [20] and posteriorly indexed to the body surface area to obtain the left ventricular mass index (LVMI). Pulse wave Doppler in the 4-chamber view was used to assess mitral inflow patterns (E/A ratio, deceleration time). Pulsed wave tissue Doppler imaging of both the lateral and septal walls was performed to measure early diastolic e tissue velocity (e′). To assess LV filling pressures we calculated E/e′ [21]. The algorithm to detect and classify the severity of LV diastolic dysfunction was based in the current recommendations for the evaluation of LV diastolic function by Echocardiography [21].

### Speckle tracking echocardiography

**Fig. 1:**
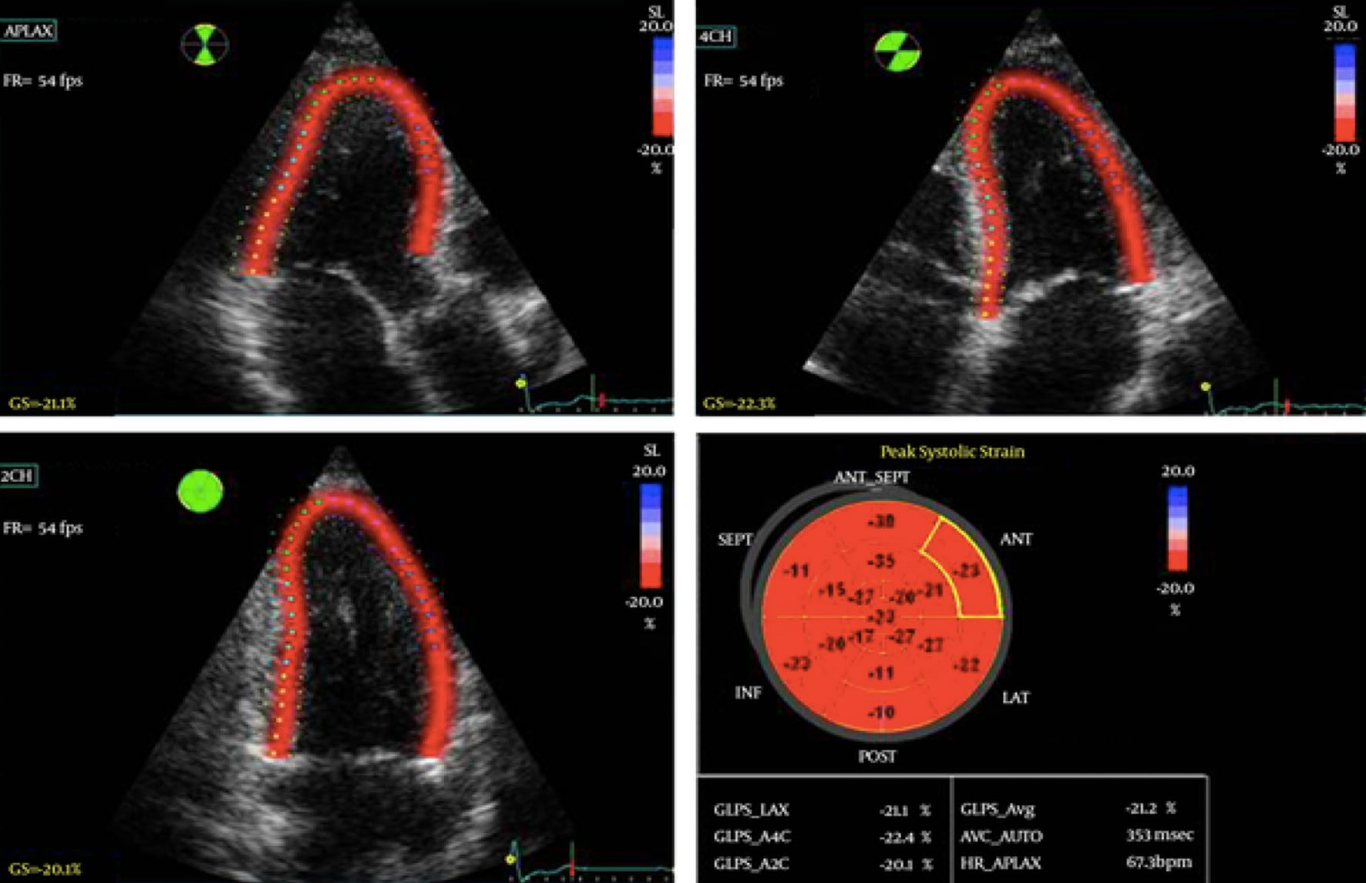
Left ventricular global longitudinal strain (GLS) in a chronic kidney disease patient who did not reach the primary endpoint (MACE). GLS represents the average value of the peak systolic longitudinal strain of the three apical views (four-chamber, two-chamber, and long-axis), using a 17-segment model. The *bottom right* graph depicts a bulls-eye plot with regional (each segment) and global (average) strain. Nearly normal strain is represented in *red*, whereas impaired strain is represented in shades of *red*/*pink*, with areas of most impaired strain depicted in very *light pink* or *blue*. In this patient LV GLS was within normal limits at −21.2%. *AVC* aortic valve closure, *APLAX* apical long axis, *2Ch* two-chamber, *4Ch* four-chamber

Customized software performed the speckle tracking analysis for VIVID 7 (2D-strain EchoPac PC v.7.0.1, GE Healthcare Little Chalfont, United Kingdom). Global longitudinal strain (GLS) Fig. 1 and strain rate (GLSr) were obtained from the three apical views. Global circumferential strain (GCS) and strain rate (GCSr) values were obtained from the basal, mid, and apical short-axis planes. Global strain values were calculated from the average of the 18 segments in the longitudinal or circumferential planes. LV twist was calculated as the net difference in peak systolic rotational strain between the six basal and six apical segments. The early and late diastolic SR parameters SRe and SRa were calculated from 18 segments in the longitudinal direction. A SRe/SRa ratio <1.1 was regarded as an index of altered segmental relaxation—segmental DD [22]. The total number of segmental DD was calculated for all participants.

### Vascular stiffness

**Fig. 2:**
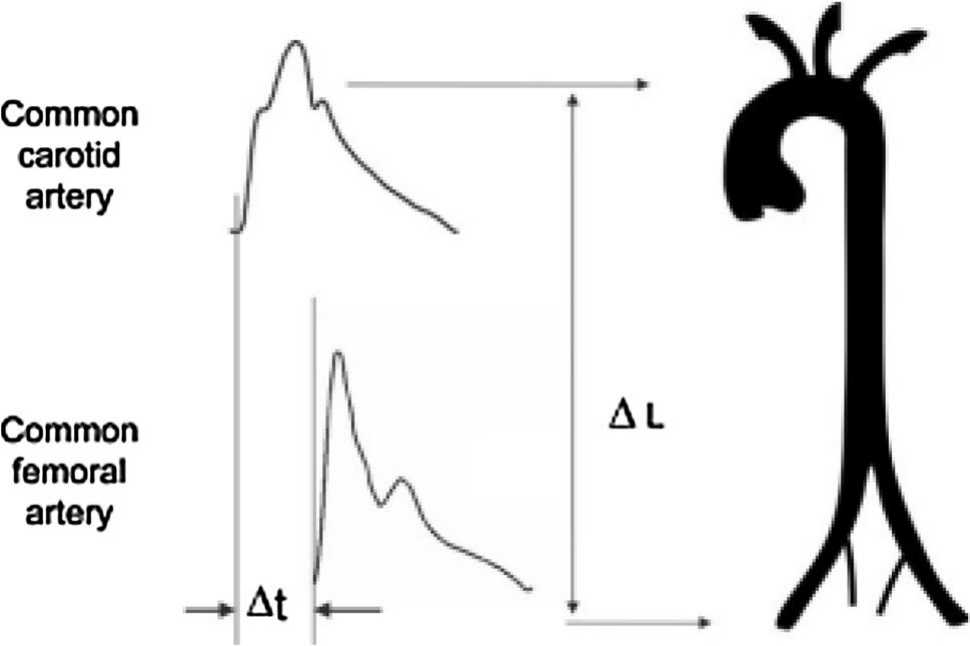
Aortic PWV was measured using the foot-to-foot velocity method from various waveforms. These are usually obtained, transcutaneously at the common carotid artery and the femoral artery (i.e. ‘carotid-femoral’ PWV − ΔL), and the time delay (Δt or transit time) measured between the feet of the two waveforms

On the same day, approximately 10 min after performing the echocardiogram, blood pressure was measured with the subjects still in a supine position. Pressure waveforms were recorded on the carotid and femoral arteries using applanation tonometry [23]. Carotid-femoral aPWV Fig. 2 was calculated using a commercially available device (SphygmoCor, Pulse Wave Analysis System, AtCor Medical), with a high-fidelity Millar strain-gauge transducer (Millar Instruments, Houston, TX) as described previously [24]. Two separate operators collected the measurements with coefficient of variation of <10%.

### Statistical analysis

SPSS V22 (IBM Corp. Armonk, NY, 2015) was used for statistical analysis. Normality of the variables assessed was tested using the Kolmogorov–Smirnov test. Data are presented as mean ± SD. Continuous variables were compared using a Student’s *t* test and categorical variables using the Chi square or Fisher’s exact test as appropriate. Estimated primary outcome free survival in different groups was calculated using the Kaplan Meier method, and comparisons were made using the log-rank test. Multivariate Cox proportional hazards regression analysis was performed to identify independent predictors of the primary outcome. Two tailed p values of <0.05 were considered statistically significant.

## Results

Following the exclusion of patients with poor endocardial definition (9) and pre-existing structural heart disease (6) at baseline echocardiography, the final patient population included 106 patients.

### Reproducibility

We previously demonstrated excellent intra and inter-observer agreement of STE and aPWV values between individuals [25].

### Baseline clinical and imaging characteristics according to the primary endpoint

Median follow up was 49 months (interquartile range 11–63 months). Overall 26 patients (24.5%) reached the primary end point. During the follow up period, five deaths (4.7%) (3 due to heart failure, one sudden death and one due to sepsis), four ST elevation MI (3.8%), two non-ST elevation MI (1.9%), three admissions for urgent PCI (2.8%), five admissions for an elective PCI (4.7%), three CABG revascularizations (2.8%), one unstable angina [leading to CABG revascularization (0.9%)], one admission for heart failure (0.9%) and two strokes (1.9%) occurred.

The baseline clinical and imaging characteristics are shown in Table 1. There were no significant differences in demographic characteristics between patients suffering MACE and those who did not. Patients who suffered MACE had higher BMI (27.5 vs. 24.3, p = 0.04), lower eGFR (23.2 vs. 47.5, p < 0.001) and a higher percentage of hypertensive heart disease (82% vs. 65% p = 0.01). Baseline GLS (−16.8% vs. −19.1%, p < 0.001) and GLSr (−0.78 vs. −1.03 m.s, p = 0.01) were significantly decreased in patients suffering MACE, while LV twist (19.3° vs. 16.3° p = 0.05) and aPWV (11.5 vs. 8.6 m/s p < 0.001) were significantly higher.

**Table 1:** Baseline demographic, clinical and imaging characteristics divided into groups according to the occurrence or not of MACE at follow-up

	Free of MACE N = 80	Suffered MACE N = 26	p value
General demographics			
Age, years	54.5 ± 13	60.3 ± 12	0.108
Male gender, *n* (%)	39 (48.7)	15 (54.3)	0.06
BSA, m^2^	1.9 ± 0.21	1.8 ± 0.23	0.854
Clinical characteristics			
BMI, kg/m^2^	24.3 ± 4.3	27.5 ± 5.2	0.040*
eGFR, mL/min/1.73 m^2^	23.2 ± 5.8	47.7 ± 9.3	<0.001*
SBP, mmHg	130.2 ± 22.1	139.6 ± 24.4	0.392
DBP, mmHg	78.4 ± 10.1	84.1 ± 13.7	0.764
Diabetes mellitus, n (%)	36 (41)	15 (54)	0.265
Hypertension, n (%)	51 (65)	23 (82)	0.01*
Urea, mg/dL	10.7 ± 2.1	16.1 ± 3.8	0.01*
Creatinine	150.6 ± 27.1	269.1 ± 31.9	<0.001*
Total Cholesterol mmol/L	4.4 ± 0.7	4.6 ± 0.6	0.463
LDL, mmol/L	2.2 ± 0.37	2.5 ± 0.42	0.22
HDL, mmol/L	1.4 ± 0.23	1.1 ± 0.20	0.138
Smoking (ever), n (%)	6 (7.9)	5 (15.6)	0.432
Conventional echocardiography			
IVSd, cm	0.98 ± 0.08	1.2 ± 0.13	0.03*
LVIDd, cm	4.4 ± 0.51	4.6 ± 0.68	0.180
LVIDs, cm	2.9 ± 0.36	3.1 ± 0.27	0.445
LVPWd, cm	0.92 ± 0.06	0.97 ± 0.08	0.191
FS, %	34.1 ± 8.1	29.7 ± 5.8	0.238
LVMI, g/m^2^	72.5 ± 16.5	88.2 ± 19.1	0.02*
RWT	0.41 ± 0.0.6	0.44 ± 0.07	0.412
Biplane LVEF, %	63.2 ± 9.2	59.1 ± 10.7	0.231
LAVI mL/m^2^	24.6 ± 3.8	32.6 ± 4.3	0.02*
E/A ratio	1.06 ± 0.09	1.10 ± 0.07	0.745
E/e′ (average)	7.1 ± 1.4	12.5 ± 1.9	0.001*
MV Dec Time, ms	221 ± 33.1	195 ± 29.4	0.672
Diastolic dysfunction, n (%)			0.001*
Type I	30 (38.4)	11 (60.1)	
Type II	2 (3.1)	2 (7.9)	
Type III	0	1 (1.1)	
Not present	46 (58.5)	14 (30.9)	
Speckle tracking echocardiography parameters			
GCS, %	−24 ± 2.08	−22.9 ± 3.11	0.228
GLS, %	−19.1 ± 3.81	−16.9 ± 3.96	0.01*
GCSRs, s^− 1^	−1.51 ± 0.13	−1.42 ± 0.17	0.231
GLSRs, s^− 1^	−1.03 ± 0.09	−0.78 ± 0.22	0.01*
LV Twist, °	16.3 ± 4.82	19.3 ± 5.30	0.05*
Number seg DD	7.1 (1.5–8)	9.9 (1.9–12.1)	0.038*
aPWV, m/s	8.6 ± 1.7	11.5 ± 2.41	0.001*

Data is presented as percentage, mean ± standard deviation or median (interquartile range) as appropriate

*BSA* body surface area, *eGFR* estimated glomerular filtration rate, *SBP* systolic blood pressure, *DBP* diastolic blood pressure, *DM* diabetes mellitus, *IVSd* intreventricular septum, *LVIDd* left ventricular internal dimension diastole, *LVIDs* left ventricular internal dimension systole, *LVPWd* left ventricular posterior wall thickness diastole, *FS* fractional shortening, *LVMI* left ventricular mass index, *RWT* regional wall thickness, *LVEF* left ventricular ejection fraction, *LAVI* left atrium volume indexed to BSA, *E/A* ratio mitral inflow peak early velocity to mitral inflow peak late velocity, *E/e*′ ratio mitral inflow peak early velocity to tissue Doppler mitral annular early velocity, *MV* mitral valve, *GCS* global circumferential strain, *GLS* global longitudinal strain, *GCSr* global circumferential strain rate, *GLS* global longitudinal strain rate, *LV* left ventricular, *DD* diastolic dysfunction, *aPWV* aortic pulse wave velocity

*Statistically significant

### Univariate and multivariate predictors of outcome

As shown in Table 2, univariate Cox proportional hazards modelling demonstrated that a higher LVMI [hazard ratio (HR) 1.078, 95% confidence interval (CI) 1.002–1.035, p = 0.027], increased E/e′ (HR 1.177, 95% CI 1.091–1.270, p < 0.001), impaired GLS (HR 1.239, 95% CI 1.088–1.410, p = 0.01) and GLSr (HR 3.369, 95% CI 1.710–6.638, p < 0.001) and a higher aPWV (HR 1.331, 95% CI 1.089–1.692, p = 0.001) were significantly associated with the primary end−point.

**Table 2:** Univariate and multivariate Cox proportional hazards regression analysis for the primary endpoint (major adverse cardiovascular events)

	Univariate analysis		Multivariable analysis	
	Hazard ratio (95% CI)	p value	Hazard ratio (95% CI)	p value
Demographics/clinical characteristics				
Age	1.02 (0.99–1.04)	0.128		
Male gender	0.56 (0.26–1.19)	0.136		
HT	2.83 (0.63–12.59)	0.172		
DM	1.66 (1.12–2.51)	0.06		
eGFR	1.10 (1.05–1.15)	0.001*	1.06 (1.02–1.16)	0.01*
Conventional echocardiographic parameters				
IVSd	7.17 (0.91–18.43)	0.083		
LVIDd	1.77 (0.79–3.38)	0.165		
LVIDs	1.43 (0.676–3.06)	0.345		
LVPWd	2.96 (0.67–13.04)	0.151		
LVMI	1.07 (1.00–1.03)	0.027*	1.02 (0.96–1.13)	0.09
RWT	7.96 (1.45–19.65)	0.351		
Biplane LVEF	0.96 (0.89–104)	0.341		
LAVI	1.73 (1.37–1.95)	0.032*	1.46 (0.94–1.13)	0.18
E/A ratio	1.97 (1.21–4.34)	0.121		
E/e′ (average)	1.17 (1.09–1.27)	<0.001*	1.13 (1.02–1.21)	0.020*
Diastolic dysfunction Grade ≥ 1	1.25 (1.08–1.35)	0.01*	1.10 (0.99–1.03)	0.11
Speckle tracking echocardiographic parameters				
GCS	1.05 (097–1.14)	0.185		
GLS	1.23 (1.08–1.41)	0.01*	1.12 (1.02–1.29)	0.041*
GCSRs	2.05 (0.61–6.86)	0.241		
GLSRs	3.36 (1.71–6.63)	0.04*	1.60 (0.55– 4.81)	0.115
LV twist	1.10 (1.03–1.18)	0.06		
Number seg DD	1.07 (0.98–1.17)	0.124		
Arterial stiffness				
aPWV	1.33 (1.08–1.69)	0.001*	1.31 (1.05–1.41)	0.021*

Data is presented in HR, hazards ratios; CI, confidence interval (95%), p value

*HT* hypertension, *DM* diabetes mellitus, *eGFR* estimated glomerular filtrarion rate, *IVSd* intraventricular septum, *LVIDd* left ventricular internal dimension diastole, *LVIDs* left ventricular internal dimension systole, *LVPWd* left ventricular posterior wall thickness diastole, *LVMI* left ventricular mass index, *RWT* regional wall thickness, *LVEF* left ventricular ejection fraction, *LA* left atrium, *E/A* ratio mitral inflow peak early velocity to mitral inflow peak late velocity, *E/e′* ratio mitral inflow peak early velocity to tissue Doppler mitral annular early velocity, *GCS* global circumferential strain, *GLS* global longitudinal strain, *GCSr* global circumferential strain rate, *GLS* global longitudinal strain rate, *LV* left ventricular, *DD* diastolic dysfunction, *aPWV* aortic pulse wave velocity

*Statistically significant

In a multivariable analysis (Table 2), GLS (HR 1.12, 95% CI 1.02–1.29, p = 0.041) and aPWV (HR 1.31, 95% CI 1.05–1.41, p = 0.021) remained significantly and independently associated with the primary end-point.

**Fig. 3:**
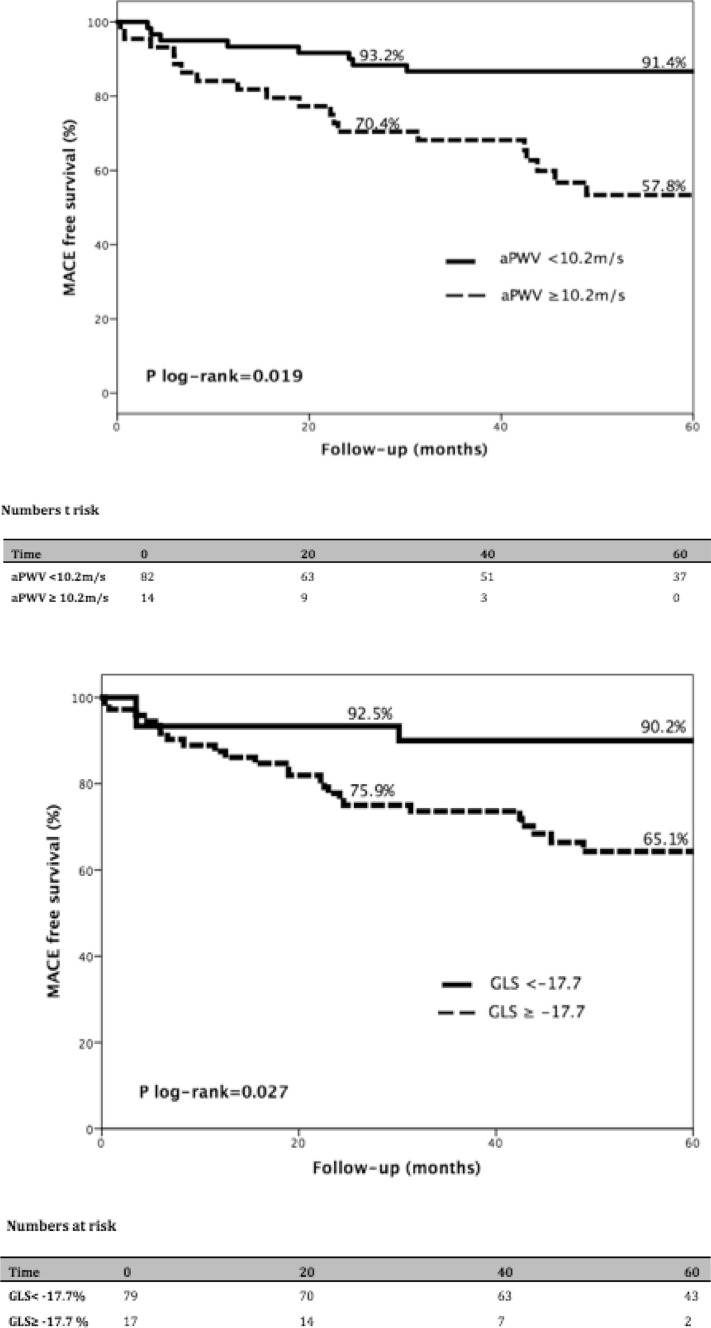
Event free survival according GLS (u*pper graph*) and aPWV (*lower graph*) cut−off values. Kaplan–Meier time to primary endpoint curves stratified according to a GLS and aPWV of −17.7% and 10.2 m/s respectively. Patients with GLS more then (less negative) −17.7% and aPWV more than 10.2 m/s were more likely to reach a primary endpoint at a median follow up of 49 months ± 9 months when compared with those with a GLS less then (more negative) −17.7% and aPWV of less then 10.2 m/s

From all the imaging parameters, we found that GLS with a cut-off value of −17.7% (72.3% sensitivity, 70.3% specificity), and aPWV with a cut-off value of 10.2 m/s (76.1% sensitivity and 69.6% specificity) had the best discriminatory power to predict the occurrence of the primary outcome (Table 3). Furthermore, patients with a GLS greater than −17.7% (log rank p = 0.027) and aPWV of 10.2 m/s or more (log rank p = 0.019) had a reduced estimated MACE free survival at 49 months follow up (Fig. 3).

**Table 3:** Cut-off values stratified according to the best sensitivity and best specificity values to predict the presence of the primary endpoint

	Cut-off value	Sensitivity (%), (95% CI)	Specificity (%), (95% CI)	Area under the curve
GLS (%)	−17.7	72.3 (57.4–81.7)	70.3 (41.3–82.6)	0.71
GCS (%)	−23.4	57.1 (43.1–69.3)	56.8 (26.3–76.5)	0.51
GLSr (s^−1^)	−0.98	67.9 (48.8–75.4)	52.7 (53.0–87.4)	0.58
GCSr (s^−1^)	−1.4	53.6 (41.3–70.3)	49.2 (46.3–64.3)	0.49
LV twist (°)	17.5	62.3 (57.5–79.4)	56.1 (53.6–88.1)	0.61
Number seg DD	7.5	64.3 (46.8–70.9)	52.4 (49.9–76.3)	0.58
aPWV (m/s)	10.2	76.1 (58.4–89.6)	69.6 (55.6–84.2)	0.74
E/e′	11.7	78.1 (66.1–89.8)	82.4 (70.2–90.1)	0.79

*CI* confidence interval, *GCS* global circumferential strain, *GLS* global longitudinal strain, *GCSr* global circumferential strain rate, *GLSr* global longitudinal strain rate, *LV* left ventricular, *DD* diastolic dysfunction, *aPWV* aortic pulse wave velocity, *E/e′* ratio mitral inflow peak early velocity to tissue Doppler mitral annular early velocity

### Sub-analysis according to CKD stages

Cox univariate regression sub-analysis between CKD groups demonstrated that more advanced CKD (stages 4 and 5) had more outcomes when compared with earlier stages CKD (17% vs. 7.5% p_log rank_ = 0.001). Aortic PWV was a significant predictor in later CKD stages (HR 1.48, 95% CI 1.03–1.79, p = 0.022). Although we didn’t find any statistical significance from the speckle tracking parameters between the groups we saw a considerable trend toward significance with GLS (HR 1.189, 95% CI 0.971–1.383, p = 0.071) Table 4.

**Table 4:** Univariate Cox proportional hazards regression sub-analysis for the primary endpoint according to CKD stages

	CKD stages 1–3		CKD stages 4, 5	
	HR CI (95%)	p value	HR CI (95%)	p value
GCS	0.99 (0.89–1.10)	0.870	1.03 (0.90–1.18)	0.640
GLS	1.09 (0.95–1.50)	0.228	1.18 (0.97–1.38)	0.071
GCSRs	1.32 (0.76–6.65)	0.739	1.88 (0.92–5.31)	0.538
GLSRs	2.01 (0.61–4.86)	0.141	1.32 (1.04–1.42)	0.112
LV Twist	1.05 (0.94–1.18)	0.356	1.04 (0.94–1.14)	0.384
Number seg. DD	0.97 (0.83–1.17)	0.716	1.06 (0.93–1.20)	0.369
aPWV	1.05 (0.88–1.24)	0.279	1.48 (1.03–1.79)	0.022*

*GCS* global circumferential strain, *GLS* global longitudinal strain, *GCSr* global circumferential strain rate, *GLSr* global longitudinal strain rate, *LV* left ventricular, *DD* diastolic dysfunction, *aPWV* aortic pulse wave velocity

*Statistically significant

## Discussion

This prospective, cohort study demonstrates that GLS and aPWV are significant, independent predictors of MACE in CKD patients with no clinical and conventional echocardiographic evidence of CVD.

Previous observational and prospective studies have demonstrated that in the general population, GLS provides incremental value over conventional imaging [26] and biochemical measurements [27] in predicting survival following MI [27], cardiac surgery [28] or heart failure [29]. Few studies have addressed the relationship between GLS and CV mortality in CKD patients. In 2014, a single-centre study by Krishnasamy et al. [13] examined the association between GLS and all-cause mortality in 447 CKD patients. In the 5-year follow-up period, GLS was a significant independent predictor of mortality (HR 1.08, 95% CI 1.01–1.15) [13]. More recently, Kramann et al. demonstrated the ability of strain parameters to identify uremic cardiomyopathy and predict CV mortality in dialysis patients [12]. Of interest, however, a large portion of the patients included in the afore-mentioned studies had already suffered a cardiovascular event, hence confounding the significance of their results in patients with no clinical cardiovascular disease. Additionally, GLS has been shown to be sensitive to loading conditions, especially afterload [30] consequently, variations in GLS measurements due to loading conditions occur in dialysis patients and results from such studies should be viewed with caution. Lastly, novel deformation parameters (i.e. LV twist or strain rate) were not assessed. Speckle tracking echocardiography allows direct assessment of myocardial muscle shortening and lengthening by assessing not only strain, but also strain rate and LV rotation/twist [31].

In the current study we used a wide range of subclinical cardiovascular measures such as strain, strain rate, LV twist, number of segments with DD or aPWV, in an attempt to identify subclinical prognostic information. Our cohort encompassed CKD patients at all stages, with no previous history of CVD, while dialysis patients were excluded. Our results demonstrated a significant, independent relationship between impaired GLS and MACE in CKD patients with no CVD and normal EF on conventional echocardiography. In our cohort a GLS of −17.7% or less had a good discriminative power (sensitive and specificity) to predict MACE. Another interesting finding of the current study is that, at baseline, patients that reached our composite primary endpoint (MACE) had significantly impaired GLS compared to patients who did not suffer MACE, even though LVEF was similar in both groups. This fact suggests that GLS may be able to detect CKD-related myocardial changes early in its course, as it primarily evaluates the function of subendocardial longitudinal fibres that are more sensitive to cardiac injury [32]. In fact, animal and human studies support the idea that changes in myocardial composition and function that are fuelled by traditional (hypertension, diabetes) and non-traditional (oxidative stress, inflammation, thrombosis) risk factors occur early in the course of the disease, prior to the onset of clinical manifestations [33, 34], supporting the hypothesis that GLS could act as a CV biomarker of outcome in the high risk cohort of CKD patients. The majority of deaths in our cohort were caused by heart failure (3/5). In patients with heart failure, GLS was shown to be a superior predictor of cardiac events and all-cause mortality compared to EF [29, 35]. GLS is impaired in heart failure patients with preserved ejection fraction (HFpEF) [36, 37]. This relates to the fact that subendocardial function is likely to be impaired in diastolic heart failure patients while epicardial/transmural function remains preserved which explains the preserved LV ejection fraction (LVEF). Abnormal longitudinal function in this cohort can be detected at an earlier stage by examining subendocardial function, by cardiac magnetic ressonace [38] or by GLS measurement [37]. This suggests that GLS could act as more sensitive prognostic biomarker for HFpEF patients.

None of the remaining deformation parameters were significant predictors of CV events. Due to the derivation of strain, SR is inherently noisier than strain, and previous correlations between MRI tagging and STE were poorer for SR when compared with strain [39], which may partly explain our findings. It is also known that GCS may remain relatively preserved to compensate for cardiac function when longitudinal function begins to become impaired in patients with early stage heart failure [40]. LV hypertrophy, which is observed even in early stage CKD [33], is mainly caused by hypertrophic responses in the mid-myocardial layers that are predominantly circumferentially orientated. This compensates for the reduction in longitudinal function and may explain preserved LVEF and GCS [41] in our cohort.

LV rotational mechanics are altered with advancing age and various medical conditions such as MI, hypertension, diabetes mellitus and dilated cardiomyopathies [42–45], however, very little is known about LV twist in CKD. The LV twists along its long axis during the cardiac cycle as the result of counter clockwise and clockwise rotation of the apex and the base, respectively, in systole and vice versa in diastole [46]. This twisting motion or torsion has been attributed to interaction between the epicardial and endocardial helical myocardial fibre couples [31]. Our group previously identified, for the first time LV twist as a subclinical marker of early renal impairment [47], nevertheless in the current study no significant independent association between twist and outcomes was identified. We found, however, that patients who suffered MACE had significantly higher LV twist, supporting the growing evidence that LV rotational mechanics can potentially be used as an early sensitive marker of myocardial disease. Larger outcome studies are required to assess whether this parameter may be useful for prediction of cardiac events in CKD patients.

From the conventional echocardiographic parameters, only E/e′ (average) was a significant, independent predictor of MACE in our cohort. LV diastolic dysfunction, characterised by abnormalities of ventricular filling, including decreased diastolic distensibility and impaired relaxation, is a very common structural abnormality in patients with CKD [48]. E/e′ has been considered a robust marker in the prediction of LV filling pressure in a variety of conditions [49] and it has been validated against invasive methods for LV filling pressure assessment [50]. Our findings are in line with previous studies suggesting that E/e′ ratio, which is elevated in CKD and ESRF patients [51], predicts mortality and cardiovascular events in CKD patients with diastolic dysfunction [52, 53].

Arterial remodelling is known to be present even in the early stages of CKD. Compared with normotensive and hypertensive controls, patients with CKD stages 2–5 had significantly larger internal carotid artery diameters but comparable intima-media thickness, resulting in significantly increased circumferential wall stress [54]. When it comes to CV events, our study complements the findings of the Nephrotest study [55] regarding the important relationship between aPWV and all cause mortality and non-fatal CV events in CKD. They identified aPWV as an independent predictor of all-cause mortality and fatal or non-fatal CV events in 439 patients with CKD stages 3–5. However, 28% of their population had a history of CV disease. The present study further extends these findings in a cohort with no clinical evidence of CVD. In our study, patients with an aPWV of 10.2 m/s or more had reduced estimated MACE free survival at median follow up of 49 months (57.8% vs. 91.4%, p_log rank_ = 0.019).

We also aimed to perform a sub analysis to assess predictors of outcome in patients with early (stages 1–3) versus advanced (stages 4,5) CKD. A few authors already established that cardiac and arterial changes start to develop early in the uremic milieu [14, 56]. In our paper, however, the absence of significant predictors of outcome in CKD stages 1–3 can be explained by the low number of outcomes in this sub-group when compared with CKD stages 4–5 (7.5% vs. 17%, p_log rank_ = 0.001). Nevertheless, in CKD stages 4–5, we found that aPWV is the most robust parameter to predict MACE in our cohort, which is in line with previous research [55]. In statistical terms, GLS was not a significant predictor in either group. Larger outcome studies are necessary to study the value of GLS within CKD groups.

The current study presents a number of strengths. It includes a comprehensive assessment of subclinical myocardial and vascular parameters in an asymptomatic from the cardiovascular point of view CKD cohort with a long follow-up period (49 ± 9 months). However the results of this study may require careful interpretation due to some limitations. (1) Although STE measurements as strain or LV twist have been validated against sonomicrometry and CMR, vendor reproducibility [57], the need for optimal image quality and time constraint are currently limiting STE use in routine clinical practice; (2) The presence of significant coronary epicardial artery disease was not ruled in all patients. All patients’ from our cohort were clinically asymptomatic with normal LVEF and no regional wall motion abnormalities by conventional echocardiography. Therefore they did not fulfill the criteria to be subjected to any invasive (coronary angiography) or functional testing. However, within our cohort in a subset of 20 pre-dialysis patients who underwent elective invasive angiography as part of their pre-transplant assessment (following recruitment to our study), the vast majority of patients (N = 19) had no significant disease in their epicardial arteries. Additionally, 12 additional CDK stage 4–5 patients had a Dobutamine Stress Echocardiogram as part of their pre transplant assessment. Eleven patients had a negative test for ischaemia and 1 patient had blunted chronothropic response and thus did not achieve 85% of maximal predicted heart rate despite the use of atropine. From a total 32 patients that underwent functional/invasive testing to exclude the presence of significant coronary/myocardial disease, 30 (94%) had no significant epicardial disease/myocardial ischemia. This fact supports the hypothesis that microvascular ischaemia may account for the GLS patterns observed in the current CKD population. Interesting to notice that despite the absence of myocardial ischaemia and normal epicardial arteries 7 of these patients suffered MACE. This is line with current literature that suggests that non-traditional risk factors—such as inflammation, oxidative stress, and elevated lipoprotein levels—have been shown to have a role in promoting CVD risk [58, 59]. (3) Even though we adjusted for a large number of patient characteristics, the possibility of residual confounding effects cannot be excluded.

## Conclusion

In CKD patients with no clinical or conventional echocardiographic evidence of significant CVD, GLS and aPWV independently predict the occurrence of MACE. If our findings are validated in larger prospective studies, GLS and aPWV may become a useful addition to the current risk stratification tools, aiming to prevent future cardiovascular events in this high-risk patient cohort.
